# Yorkshire Terriers under primary veterinary care in the UK – demography and disorders

**DOI:** 10.1186/s40575-025-00145-y

**Published:** 2025-08-13

**Authors:** Dan G. O’Neill, Sara D. Witkowska, Dave C. Brodbelt, David B. Church, Karolina S. Engdahl

**Affiliations:** 1 Pathobiology and Population Sciences, The Royal Veterinary College, Hawkshead Lane, North Mymms, Hatfield, AL9 7TA Herts UK; 2 Clinical Science and Services, The Royal Veterinary College, Hawkshead Lane, North Mymms, Hatfield, AL9 7TA Herts UK; 3 Department of Clinical Sciences, Swedish University of Agricultural Sciences, PO Box 7054, Uppsala, 750 07 Sweden

**Keywords:** VetCompass, Electronic health record, EHR, Breed, Dog, Epidemiology, Primary-care, Veterinary, Pedigree, Yorkshire Terrier

## Abstract

**Background:**

The Yorkshire Terrier is a long-established and commonly owned dog breed. This study aimed to explore anonymised primary-care veterinary clinical data from the VetCompass Programme to characterise the demography, common disorders and longevity of the general population of Yorkshire Terriers in the UK in 2016.

**Results:**

Yorkshire Terriers composed 28,032 (3.10%) of the study population of 905,542 dogs under veterinary care in 2016. Annual proportional birth rates decreased in popularity between 2005 and 2016, from 3.54% of all dogs born in 2005 to 2.15% in 2016. The median adult bodyweight was 5.06 kg (IQR 3.81–6.49, range 1.01-15.00). Clinical records from a random sample of 3,308/28,032 (11.80%) Yorkshire Terriers were manually reviewed to extract information on all disorders diagnosed during 2016. The most commonly diagnosed disorders were periodontal disease (21.10%, 95% CI: 19.71–22.49), overgrown nail(s) (6.47%, 95% CI: 5.63–7.31), anal sac impaction (3.99%, 95% CI: 3.32–4.66), overweight/obesity (3.72%, 95% CI: 3.07–4.36) and persistent deciduous teeth (3.57%, 95% CI: 2.94–4.20). Among the 464/3,308 (14.03%) Yorkshire Terriers that died during the study period, the median age at death was 13.56 years (IQR 11.30–15.15, range 0.06–19.08). The most common disorder groups causing death were brain disorders (9.79%, 95% CI: 6.79–12.78) and kidney disorders (8.73%, 95% CI: 5.88–11.58).

**Conclusions:**

The current study identifies a reducing ownership trend for Yorkshire Terriers in the UK. Disorders that are common and appear predisposed in Yorkshire Terriers include periodontal disease, persistent deciduous teeth and patellar luxation. Overall, the Yorkshire Terrier has high longevity, which is suggestive of robust overall health.

## Abstract (plain language summary)

The Yorkshire Terrier breed, colloquially known as the Yorkie, was developed and subsequently fixed as a distinct breed in approximately 1865 and was historically very common among the pedigree subset of UK dogs registered with the Kennel Club (KC). However, annual KC registrations suggest that the breed has reduced markedly in popularity more recently. The breed is widely considered to be relatively healthy and long-lived. This study aimed to explore anonymised primary-care veterinary clinical records from the VetCompass Programme to report the demographics, common disorders and longevity of the general population of Yorkshire Terriers in the UK in 2016.

Yorkshire Terriers comprised 28,032 (3.10%) of the 905,542 dogs included in the study. The percentage of all dogs born each year that were Yorkshire Terrier decreased from 3.54% of all dogs born in 2005 to 2.15% in 2016. The average bodyweight of adult Yorkshire Terriers was 5.06 kg.

The veterinary clinical records of a random sample of 3,308/28,032 (11.80%) Yorkshire Terriers were read, and information on all disorders during 2016 was extracted. The most common disorders of Yorkshire Terriers were periodontal (dental) disease (21.10%), overgrown nail(s) (6.47%), anal sac impaction (3.99%), overweight/obesity (3.72%) and retained milk (deciduous) teeth (3.57%).

Among the 464/3,308 (14.03%) Yorkshire Terriers that died during the study period, the average lifespan of Yorkshire Terriers was 13.56 years. The most common reasons for death were brain disorders (9.79%) and kidney disorders (8.73%).

Conclusions.

The current study confirmed declining ownership of Yorkshire Terriers in the UK. Disorders that are common and appear predisposed in Yorkshire Terriers include dental (periodontal) disease, persistent milk (deciduous) teeth and slipping kneecap (patellar luxation). Overall, Yorkshire Terrier has high longevity, which is suggestive of robust overall health.

## Background

The Yorkshire Terrier breed was developed and subsequently fixed as a distinct phenotype in approximately 1865 as an outcross from the Black and Tan Terrier, with contributions from the Maltese Terrier and the Skye Terrier, when traditional terriers were brought from Scotland to Yorkshire by labourers moving there to work in mines and cotton mills [1]. Originally bred as a ratting terrier, the breed soon found favour as a ‘lady’s dog’ and then also in the show ring, where the Yorkshire Terrier is the only breed still exhibited on a decorative box in the show ring [1]. The Kennel Club (KC) breed standard states a maximum bodyweight of 3.2 kg for the breed [2]. The breed, also colloquially known as Yorkie, was historically very common among the pedigree subset of dogs registered in the UK with the KC, but over recent years, it appears to have reduced markedly in popularity, with annual KC registrations decreasing by a factor of five over the past decade from 2,077 (0.93% of all registrations) in 2013 to 495 (0.18% of all registrations) in 2022 [3]. Given this progressive decline in registrations, the subset of Yorkshire Terriers that are registered with the KC is likely to soon be considered a vulnerable native breed, defined as a native British or Irish dog breed with fewer than 300 annual KC registrations [4]. However, Yorkshire Terriers appear to remain common in the wider UK dog population, with a demographic study of over two million dogs under primary veterinary care in 2019 reporting that Yorkshire Terriers are the 10th most common breed and represent 2.4% of all dogs of all ages in the UK [5]. In that same study, however, Yorkshire Terrier was only the 16th most common breed aged under one year, representing 1.5% of all dogs aged under one year, suggesting that their popularity in the general population may also be waning, although less steeply. However, the reported long lifespans of Yorkshire Terriers may partially explain the proportional differences between the two age groups, whereby relatively fewer new puppies need to be added to maintain overall population numbers for long-lived breeds than for shorter-lived breeds such as French Bulldogs [6].

The UK KC currently recommends no additional health screening schemes or DNA tests for the Yorkshire Terrier outside of standard advice to produce puppies with an inbreeding coefficient that is either at or below the breed average and ideally as low as possible [1]. Furthermore, the KC Breed Watch system currently assigns the Yorkshire Terrier as a Category 1 breed, with no points of concern specific to this breed identified for special attention by judges [7]. Despite this, there is a substantial evidence base supporting several health problems associated with the Yorkshire Terrier breed. A 2018 review of overall predispositions across all dog breeds regardless of KC status identified the Yorkshire Terrier with 47 disorder predispositions, making it the breed with the eigth highest number of predispositions among all the breeds assessed [8]. The Yorkshire Terrier has also been reported to have 15 non-conformation-linked and 11 conformation-related inherited disorders [9, 10]. These contrasting inferences from the published literature and the current KC recommendations suggest the value of a deeper understanding of the health of the current overall population of Yorkshire Terriers in the UK.

Population longevity is widely accepted in human demography as a useful proxy indicator of the general health status of a population. Low longevity implies that a high frequency of adverse events, such as disorders contributing to mortality, occur earlier in life and therefore suggests a generally less healthy population, while the converse applies equally [11]. A similar approach has been taken for dogs, whereby life tables were constructed on the basis of age at death data extracted from anonymised veterinary clinical records in the UK and were used to infer the summary health of the comparative populations of breeds [6]. Across the 18 common dog breeds assessed in that study, Yorkshire Terrier had the second highest life expectancy from the first year of life (12.54 years), outlived only by the Jack Russell Terrier at 12.72 years. Toy or small dogs are recognised to have average longer life expectancy than larger dogs, possibly related to higher rates of glycolytic metabolism and DNA damage in larger dogs, which may partially explain the relatively high longevity of the Yorkshire Terrier [12–15]. However, despite their small body size, high longevity suggests that the Yorkshire Terrier has broadly good overall health, although further study is warranted to clarify the true state of health of the breed.

There is a growing acceptance of the value of primary-care veterinary records as a useful research resource to improve the understanding of companion animal health [16–18]. In the UK, VetCompass was established in 2010 as a real-time surveillance system that applies secondary use of anonymised veterinary clinical records to investigate and report on health issues of companion animals under veterinary care [19]. With this background, the current study aimed to explore anonymised primary-care veterinary clinical data from the VetCompass Programme to characterise the demography, common disorders and longevity of the general population of Yorkshire Terriers under primary veterinary care in the UK in 2016. A specific focus was placed on exploring associations between sex of the dog and disorder risk. These results hopefully can strengthen the evidence base available to dog welfare stakeholders responsible for the progression of health and welfare programmes for Yorkshire Terriers. Specifically, dog owners and veterinary professionals may recognise common problems in these dogs earlier when given greater prior awareness of disorder risk, and kennel clubs may apply this new information for better breed health protection plans [20].

## Materials and methods

The study population included all dogs under primary veterinary care at clinics participating in the VetCompass Programme during 2016. Dogs under veterinary care were defined as those with either (a) at least one electronic health record [EHR] (free-text clinical note, treatment, or bodyweight) recorded during 2016 or (b) at least one such EHR recorded during both 2015 and 2017. The VetCompass data fields available for the current study included fixed variables of unique animal identifier, species, breed, date of birth, sex and neuter status along with time-varying variables of bodyweight, free-form text clinical notes and treatment with relevant dates.

Dogs recorded as Yorkshire Terriers were categorised as Yorkshire Terriers, whereas all remaining dogs were categorised as non-Yorkshire Terriers. The bodyweight, sex, neuter status, and age of Yorkshire Terriers under veterinary care during 2016 were described. *All-age Bodyweight* (kg) described all available bodyweight and date combinations. *Adult Bodyweight* (kg) described the mean bodyweight recorded from all bodyweight data for dogs aged over 18 months and was categorised into 5 groups (< 3, 3 to < 5, 5 to < 7, 7 to < 9, ≥ 9). *Neuter* described the status of the dog (entire or neutered) at the final EHR. *Age* (years) describes the age at the final date of veterinary care in 2016 (December 31st, 2016) and was categorised into 5 groups (< 3.0, 3.0 to < 6.0, 6.0 to < 9.0, 9.0 to < 12.0 and ≥ 12.0).

A cohort study design followed the EHRs from 2016 to estimate the one-year period prevalence of the most diagnosed disorders of Yorkshire Terrier dogs from a population of 905,542 dogs across all breeds under primary veterinary care during 2016 at VetCompass participating practices. Sample size calculations estimated that 3,012 dogs were needed to report frequency for a disorder with 2.0% expected prevalence, 95% confidence level, and 0.50% margin of error [21]. Ethical approval was given by the RVC Social Science Research Ethical Review Board (SSRERB) (reference number SR2018-1652).

The EHRs of a random sample from all available Yorkshire Terriers were manually reviewed in detail to extract the most definitive diagnoses recorded for all disorders recorded as existing during 2016 and to link these to the most appropriate VeNom term as previously described [22]. The extracted diagnosis terms were mapped to a dual hierarchy of precision for analysis: fine-level precision and grouped-level precision [22]. The fine-level precision terms described the original extracted terms at the maximal diagnostic precision recorded within the clinical notes (e.g., *inflammatory bowel disease* remained as *inflammatory bowel disease*). The grouped-level precision terms mapped the original diagnosis terms to a general level of diagnostic precision (e.g., *inflammatory bowel disease* mapped to *enteropathy)*. Disorders described within the clinical notes using presenting sign terms (e.g., ‘vomiting’ or ‘vomiting and diarrhoea’) without a formal clinical diagnostic term were included using the first sign listed (e.g., vomiting). Elective (e.g., neutering) or prophylactic (e.g., vaccination) clinical events were excluded. No distinction was made between preexisting and incident disorder presentations. Mortality data (recorded cause, date and method of death) were extracted for all deaths at any date during the available EHRs.

Following data checking for internal validity and cleaning in Excel (Microsoft Office Excel 2013, Microsoft Corp.), analyses were conducted via R version 4.2.1 [23]. Annual proportional birth rates described the relative proportion of Yorkshire Terrier compared with all dogs from the cohort under veterinary care in 2016 born each year from 2005 to 2016. The figure illustrating annual proportional birth rates was generated with the R package ggplot2 [24]. All bodyweight data with their associated dates at any dog age were used to generate individual bodyweight growth curves for male and female Yorkshire Terriers by plotting age-specific bodyweights overlaid with a cross medians line via the R package ggplot2 [24].

One-year (2016) period prevalence values were reported along with 95% confidence intervals (CIs) that described the probability of diagnosis at least once during 2016. The CI estimates were derived from standard errors based on approximation to the normal distribution (Wald CI) for disorders with ten or more events [25] or the Wilson approximation method for disorders with fewer than ten events [26], using the binom.approx() and binom.wilson() functions from the R package epitools [27]. Prevalence values were reported overall and separately for males and females. The median age (in years) as defined above was reported for each of the most common diagnoses at the fine-level and group-level. The 10 most common disorders at group-level precision in each of three age bands (< 3 years, 3–10 years, and > 10 years) were identified, and the prevalence of each of these disorders through life up to the age of 16 years was presented via loess curves in a figure generated with the R packages ggplot2, cowplot, and ggpubr [24, 28, 29]. A combination of the Shapiro‒Wilk test and visual assessment of histograms was used to assess the normality of continuous variables. The two-proportion z test was used to compare proportions, the chi-square test was used to compare categorical variables, and the Mann‒Whitney U test was used to compare continuous variables as those that deviated from normality [25]. Statistical significance was set at the 5% level.

## Results

### Demography

**Fig. 1:**
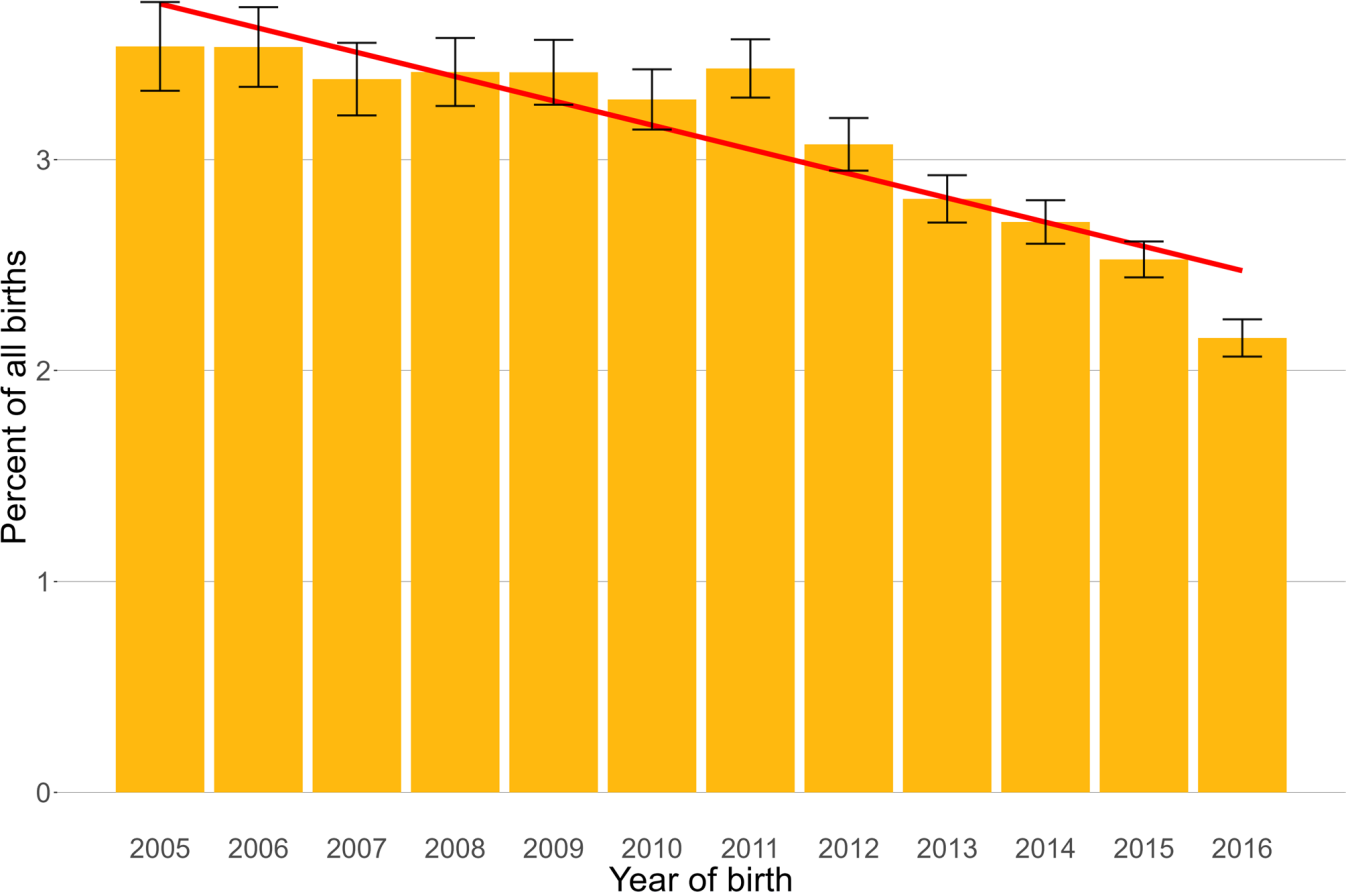
Annual proportional birth rates (2005–2016) with linear trend and 95% confidence intervals for Yorkshire Terriers (*n =* 28,032) among all dogs (*n =* 905,542) under UK primary veterinary care from January 1st to December 31st, 2016, at practices participating in the VetCompass™ Programme

The study population of 905,542 dogs under veterinary care during 2016 in the UK included 28,032 (3.10%) Yorkshire Terriers. Among the Yorkshire Terriers for whom information was available, 13,506 (48.32%) were female, and 15,974 (56.98%) were neutered (Table 1). More females than males were neutered; 58.35% of the females and 55.98% of the males were neutered (chi-square test: *P* < 0.001). The overall median age was 5.52 years (interquartile range (IQR) 2.52–9.29, range 0.02–21.60). Annual proportional birth rates for Yorkshire Terrier decreased from 3.54% of all dogs born in 2005 to 2.15% in 2016 (Fig. 1).

**Fig. 2:**
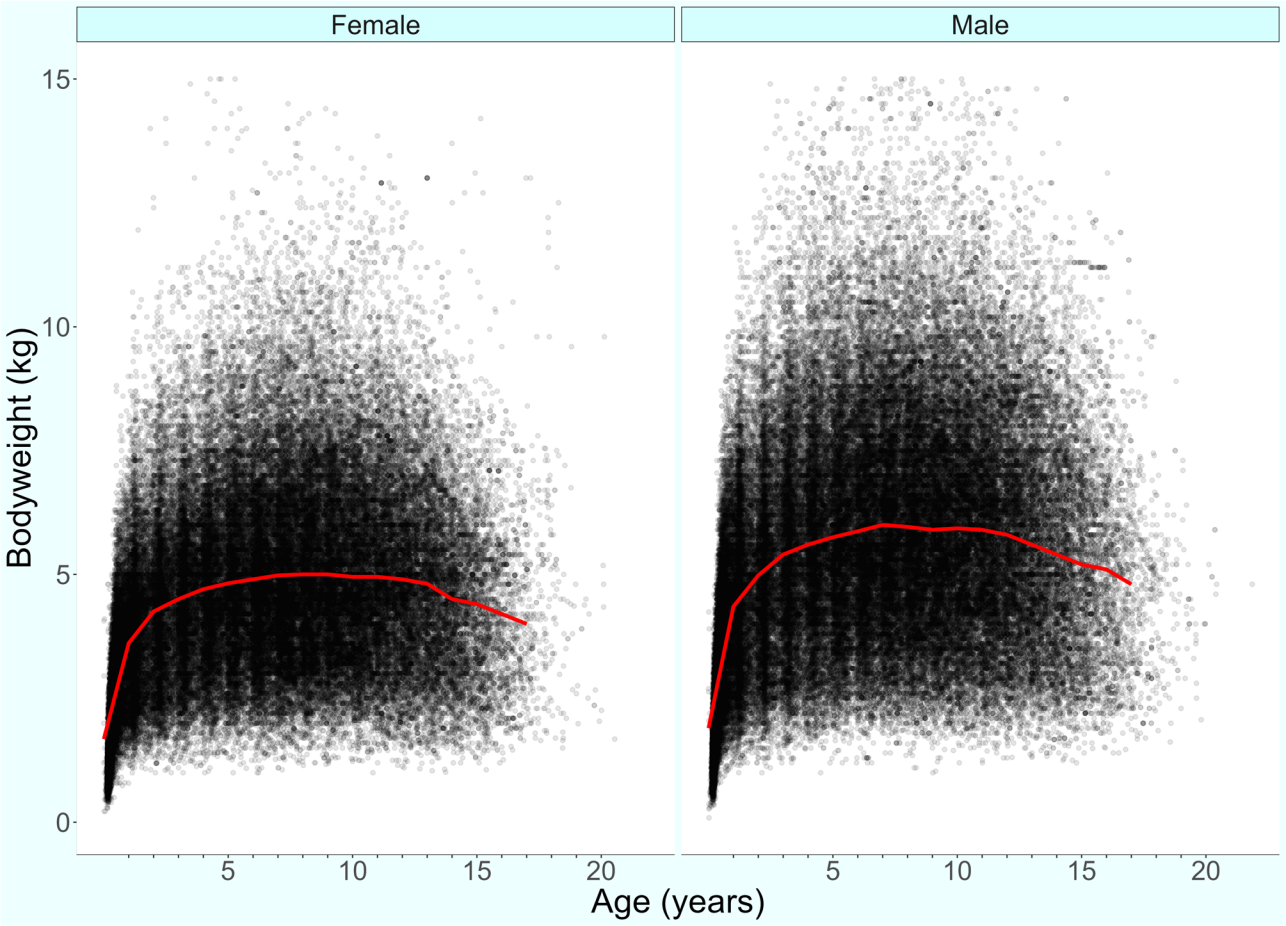
Bodyweight at different life stages with a cross-median line plot for female (*n =* 11,242) and male (*n =* 12,071) Yorkshire Terriers under UK primary veterinary care from January 1st to December 31st, 2016, at practices participating in the VetCompass™ Programme

The median adult bodyweight was 5.06 kg (IQR 3.81–6.49, range 1.01-15.00). Males (5.50 kg, IQR 4.14–6.99, range 1.03–14.60) were heavier than females (4.67 kg, IQR 3.55–5.90, range 1.01–15.00) (Mann‒Whitney U test: *P* < 0.001). The median bodyweight across all ages was also greater in males (4.96, IQR 3.54–6.49, range 0.09–14.60) than in females (4.20, IQR 3.04–5.50, range 0.28–14.60) (Mann‒Whitney U test: *P* < 0.001). Bodyweight curves based on 110,897 bodyweight values for 12,071 males and 100,854 bodyweight values for 11,242 females revealed that Yorkshire Terriers grow rapidly during their first year and continue to gain weight until approximately five years of age (Fig. 2). The proportional completeness for each demographic variable was: sex 99.72%, neuter 100.00%, mean adult bodyweight 75.22% and age 99.13%.

**Table 1:** Demography of 28,032 Yorkshire Terriers under primary veterinary care at practices participating in the VetCompass™ Programme in the UK from January 1st to December 31st, 2016. *Counts cover dogs with available data

Variable	Category	Overall No. (%)*	Female No. (%)*	Male No. (%)*
Neuter status	Neutered	15,974 (56.98)	7,881 (58.35)	8088 (55.98)
Adult bodyweight (kg)	< 3	2,406 (11.44)	1,467 (14.49)	935 (8.59)
	3 to < 5	7,809 (37.12)	4,350 (42.96)	3,451 (31.69)
	5 to < 7	6,949 (33.03)	3,152 (31.13)	3,788 (34.78)
	7 to < 9	2,978 (14.15)	969 (9.57)	2,006 (18.42)
	≥ 9	898 (4.27)	188 (1.86)	710 (6.52)
Age (years)	< 3	8,106 (29.17)	3,880 (29.02)	4,189 (29.19)
	3 to < 6	6,851 (24.65)	3,324 (24.86)	3,517 (24.51)
	6 to < 9	5,449 (19.61)	2,621 (19.60)	2,821 (19.66)
	9 to < 12	3,804 (13.69)	1,828 (13.67)	1,969 (13.72)
	≥ 12	3,579 (12.88)	1,717 (12.84)	1,855 (12.93)

### Disorder prevalence

The EHRs from a random sample of 3,308/28,032 (11.80%) Yorkshire Terriers were manually reviewed to extract information on disorders during 2016. Among these 3,308 Yorkshire Terriers, 2,157 (65.21%) had at least one disorder recorded during 2016, whereas the remainder received only prophylactic care or no active veterinary care during 2016. There were 4,212 unique disorder events reported during 2016. The median overall annual disorder count per Yorkshire Terrier was 1 (IQR 0–2, range 0–12). The distribution of annual disorder counts differed significantly between females (median count 1, IQR 0–2, range 0–9) and males (median count 1, IQR 0–2, range 0–12) (Mann‒Whitney U test, *P* = 0.002), with a lower distribution of counts in female dogs.

The 4,212 disorder events were spread across 356 fine-level disorder terms. The most commonly diagnosed disorders were periodontal disease (*n* = 698, prevalence 21.10%, 95% CI: 19.71–22.49), overgrown nail(s) (214, 6.47%, 95% CI: 5.63–7.31), anal sac impaction (132, 3.99%, 95% CI: 3.32–4.66), overweight/obesity (123, 3.72%, 95% CI: 3.07–4.36) and persistent deciduous tooth (118, 3.57%, 95% CI: 2.94–4.20). Among the 35 most common fine-level disorders, females had a greater probability of diagnosis with mammary and skin masses, whereas males had a greater probability of diagnosis with periodontal disease, aggression, heart murmur, claw injury, and cryptorchidism (two-proportion z test: *P* < 0.05). The median age of the dogs with the 35 most common fine-level diagnoses varied from 1.79 years for persistent deciduous teeth to 13.00 years for cataracts (Table 2).

**Table 2:** Prevalence of the most diagnosed disorders at a *fine-level diagnostic precision* in Yorkshire Terriers (*n* = 3,308) under primary veterinary care at practices participating in the Vet Compass Programme in the UK from January 1st to December 31st, 2016. *CI confidence interval. ** Two-proportion z test comparing female and male prevalence with *P*-values < 0.05 shown in bold

Fine-level disorder	No.	Prevalence % (95% CI*)	Female % prevalence	Male % prevalence	*P*-value**	Median age (years) of affected dogs
Periodontal disease	698	21.10 (19.71–22.49)	18.78	23.12	**0.003**	8.61 (1.30–19.27)
Overgrown nail(s)	214	6.47 (5.63–7.31)	6.54	6.42	0.944	5.36 (0.20–17.94)
Anal sac impaction	132	3.99 (3.32–4.66)	3.30	4.60	0.070	7.19 (0.84–17.71)
Overweight/obesity	123	3.72 (3.07–4.36)	4.34	3.18	0.097	7.00 (1.35–16.11)
Persistent deciduous tooth	118	3.57 (2.94–4.20)	3.56	3.58	> 0.999	1.79 (0.30–8.31)
Patellar luxation	113	3.42 (2.80–4.03)	3.63	3.24	0.605	6.18 (0.91–16.04)
Otitis externa	109	3.30 (2.69–3.90)	3.17	3.41	0.779	6.56 (0.33–16.28)
Diarrhoea	103	3.11 (2.52–3.71)	2.72	3.47	0.258	6.47 (0.33–17.70)
Dental disorder	97	2.93 (2.36–3.51)	2.40	3.41	0.106	5.07 (0.49–14.99)
Aggression	83	2.51 (1.98–3.04)	1.49	3.41	**0.001**	6.69 (0.98–16.56)
Flea infestation	82	2.48 (1.95–3.01)	2.01	2.90	0.126	4.36 (0.36–16.60)
Vomiting	82	2.48 (1.95–3.01)	2.91	2.10	0.166	3.97 (0.50–16.40)
Cataract	69	2.09 (1.60–2.57)	2.01	2.16	0.856	13.00 (4.21–18.72)
Heart murmur	67	2.03 (1.55–2.51)	1.04	2.90	**< 0.001**	12.79 (1.45–19.27)
Conjunctivitis	48	1.45 (1.04–1.86)	1.30	1.59	0.574	7.63 (1.16–14.91)
Claw injury	44	1.33 (0.94–1.72)	0.78	1.82	**0.014**	5.46 (1.11–17.82)
Pruritus	44	1.33 (0.94–1.72)	1.49	1.19	0.555	5.55 (0.52–14.48)
Gastroenteritis	42	1.27 (0.89–1.65)	0.84	1.65	0.057	3.82 (0.52–15.25)
Papilloma	38	1.15 (0.79–1.51)	1.10	1.19	0.933	12.19 (3.12–16.78)
Allergic skin disorder	37	1.12 (0.76–1.48)	1.30	0.97	0.464	6.75 (0.83–13.45)
Colitis	36	1.09 (0.73–1.44)	0.91	1.25	0.435	5.06 (0.59–14.48)
Skin cyst	36	1.09 (0.73–1.44)	1.10	1.08	> 0.999	9.51 (2.06–16.60)
Lameness	35	1.06 (0.71–1.41)	0.84	1.25	0.331	6.78 (0.53–17.46)
Osteoarthritis	33	1.00 (0.66–1.34)	1.04	0.97	0.978	12.77 (6.06–16.65)
Pyoderma	33	1.00 (0.66–1.34)	0.97	1.02	> 0.999	7.20 (1.49–17.71)
Coughing	32	0.97 (0.63–1.30)	0.58	1.31	0.052	7.23 (0.49–18.72)
Dermatitis	31	0.94 (0.61–1.27)	1.04	0.85	0.714	6.95 (2.04–16.33)
Mammary mass	31	0.94 (0.61–1.27)	2.01	0.00	**< 0.001**	12.41 (4.04–17.19)
Tracheal collapse	31	0.94 (0.61–1.27)	0.78	1.02	0.576	10.34 (1.37–16.60)
Seizure disorder	30	0.91 (0.58–1.23)	0.78	0.97	0.694	11.68 (2.36–17.49)
Gastritis	29	0.88 (0.56–1.19)	0.91	0.85	> 0.999	4.15 (0.84–14.16)
Skin mass	28	0.85 (0.53–1.16)	1.23	0.51	**0.039**	9.66 (0.97–16.33)
Wound	28	0.85 (0.53–1.16)	0.91	0.80	0.874	8.18 (2.39–15.24)
Diabetes mellitus	26	0.79 (0.49–1.09)	1.04	0.57	0.186	10.83 (7.54–14.43)
Cryptorchidism	24	0.73 (0.44–1.01)	0.00	1.36	**< 0.001**	2.10 (0.34–8.79)

The fine-level disorder terms were condensed into 71 group-level disorder terms. The most common group-level disorders were dental disorder (*n* = 888, prevalence 26.84%, 95% CI: 25.33–28.35), enteropathy (330, 9.98%, 95% CI: 8.95-11.0), skin disorders (307, 9.28%, 95% CI: 8.29–10.27), claw/nail disorders (275, 8.31%, 95% CI: 7.37–9.25) and ophthalmological disorders (219, 6.62%, 95% CI: 5.77–7.47). Among the 20 most common group-level disorders, females had a greater probability of three disorders, namely, mass, endocrine, and female reproductive disorders, whereas males had a greater probability of six disorders, namely, dental, anal sac, behavioural, upper respiratory tract, heart, and male reproductive disorders (*P* < 0.05, two-proportion z test). The median age of the dogs with the most common group-level disorders varied from 3.23 years for male reproductive disorders to 12.80 years for heart disorders (Table 3).

**Table 3:** Prevalence of the most diagnosed disorders at the *group level diagnostic precision* in Yorkshire Terriers (*n* = 3,308) under primary veterinary care at practices participating in the VetCompass™ Programme in the UK from January 1st to December 31st, 2016. *CI confidence interval. ** Two-proportion z test comparing female and male prevalence with *P*-values < 0.05 shown in bold

Group-level disorder	No.	Prevalence % (95% CI*)	Female % prevalence	Male % prevalence	*P*-value**	Median age (years) of affected dogs
Dental disorder	888	26.84 (25.33–28.35)	24.29	29.09	**0.002**	7.30 (0.30–19.27)
Enteropathy	330	9.98 (8.95-11.00)	9.00	10.85	0.085	5.01 (0.33–17.70)
Skin disorder	307	9.28 (8.29–10.27)	9.59	8.98	0.597	6.60 (0.35–18.72)
Claw/nail disorder	275	8.31 (7.37–9.25)	7.97	8.64	0.519	5.46 (0.20–17.94)
Ophthalmological disorder	219	6.62 (5.77–7.47)	6.61	6.65	> 0.999	10.58 (0.53–18.72)
Musculoskeletal disorder	203	6.14 (5.32–6.95)	5.57	6.65	0.221	8.00 (0.53–18.72)
Anal sac disorder	147	4.44 (3.74–5.15)	3.56	5.23	**0.025**	7.13 (0.84–17.71)
Behavioural disorder	129	3.90 (3.24–4.56)	3.04	4.66	**0.021**	6.00 (0.70–17.94)
Upper respiratory tract disorder	129	3.90 (3.24–4.56)	2.91	4.72	**0.009**	7.43 (0.49–18.72)
Ear disorder	125	3.78 (3.13–4.43)	3.56	3.98	0.589	6.27 (0.33–16.33)
Overweight/obesity	123	3.72 (3.07–4.36)	4.34	3.18	0.098	7.00 (1.35–16.11)
Parasite infestation	118	3.57 (2.94–4.20)	3.04	4.03	0.149	4.56 (0.36–16.60)
Neoplasia	116	3.51 (2.88–4.13)	3.63	3.41	0.813	10.61 (2.06–16.78)
Heart disorder	109	3.30 (2.69–3.90)	2.20	4.26	**0.001**	12.80 (0.39–19.27)
Mass	107	3.23 (2.63–3.84)	4.73	1.93	**< 0.001**	10.88 (0.97–17.19)
Traumatic injury	72	2.18 (1.68–2.67)	1.94	2.39	0.449	6.44 (0.62–17.83)
Brain disorder	54	1.63 (1.20–2.06)	1.42	1.76	0.526	11.68 (1.57–17.53)
Endocrine disorder	44	1.33 (0.94–1.72)	1.88	0.85	**0.016**	10.96 (6.67–15.95)
Female reproductive disorder	42	1.27 (0.89–1.65)	2.66	0.06	**< 0.001**	4.30 (0.97–16.30)
Male reproductive disorder	33	1.00 (0.66–1.34)	0.00	1.88	**< 0.001**	3.23 (0.34–19.27)

**Fig. 3:**
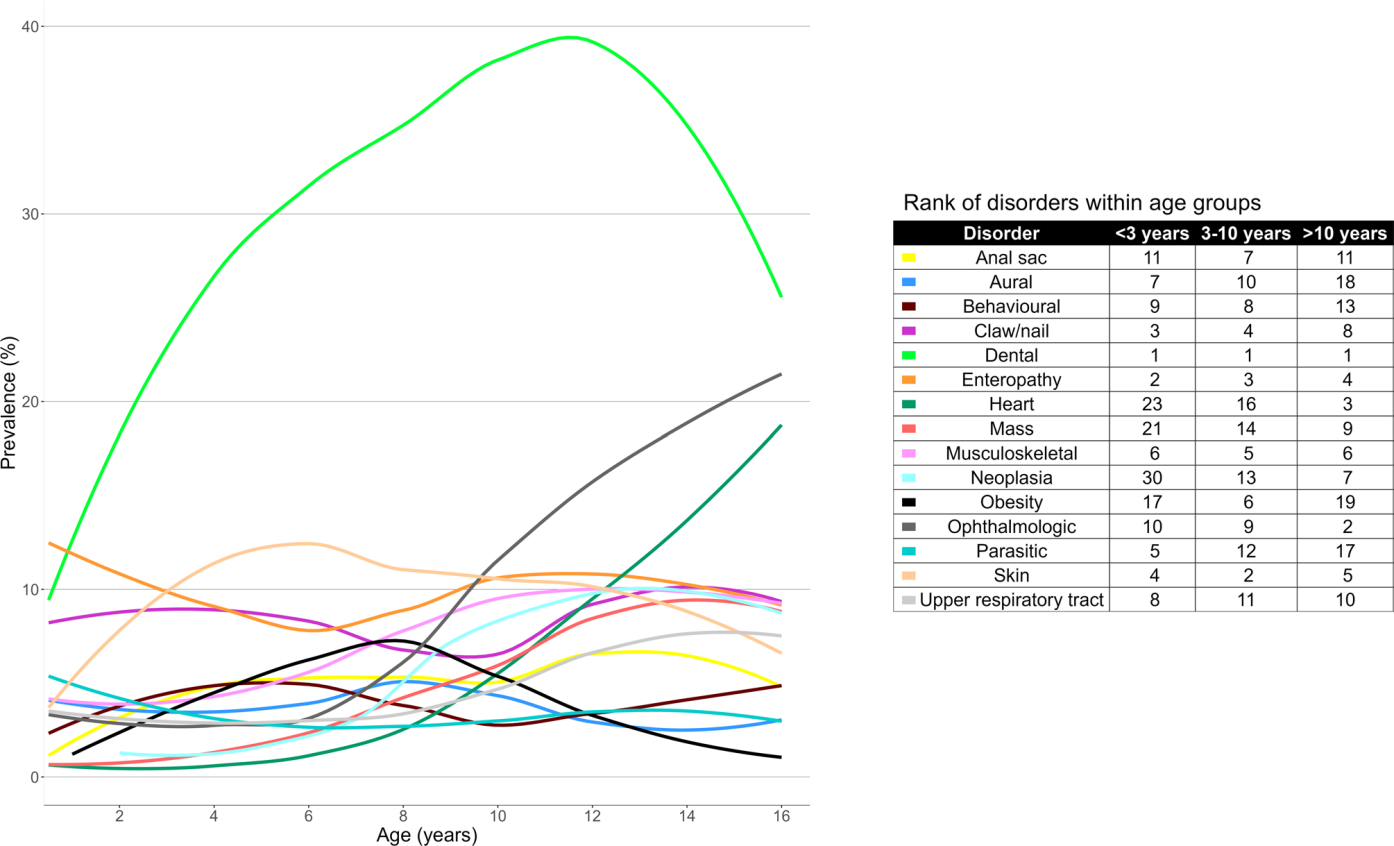
Prevalence of the 10 most commonly diagnosed group-level disorders within each of three age bands (under 3 years *n* = 930, 3–10 years *n =* 1,612, over 10 years *n* = 737) in Yorkshire Terriers under primary veterinary care at UK practices participating in the VetCompass™ Programme from January 1st to December 31st, 2016

The prevalence of the 10 most commonly diagnosed group-level disorders across three age bands: < 3 years (*n* = 930), 3–10 years (*n* = 1,612), and > 10 years (*n* = 737), is presented in Fig. 3. The prevalence of mass, obesity, anal sac, dental, heart, musculoskeletal, neoplastic, ophthalmological, skin, and upper respiratory tract disorders (10/15 of the disorders, 66.67%) varied significantly between the age groups (chi-square test, *P* < 0.05).

### Mortality

During the study period, deaths were recorded for 464/3,308 (14.03%) Yorkshire Terriers. The median age at death was 13.56 years (IQR 11.30–15.15, range 0.06–19.08). The longevity did not differ significantly between females (median longevity 13.57 years, IQR 11.26–14.92, range 0.06–19.08, *n* = 211) and males (13.50 years, IQR 11.33–15.40, range 0.21–19.06, *n* = 253) (Mann‒Whitney U test, *P* = 0.797). Among the 441/464 (95.04%) deaths with a recorded method of death, 378 (85.71%) were euthanised, and 63 (14.29%) died unassisted.

Among 378/464 (81.47%) deaths with a reported biomedical cause, the most common causes of death at the group-level precision were brain disorders (*n* = 37, 9.79%, 95% CI: 6.79–12.78), kidney disorders (33, 8.73%, 95% CI: 5.88–11.58), and appetite disorders (31, 8.20%, 95% CI: 5.44–10.97) (Table 4).

**Table 4:** Mortality in Yorkshire Terriers with a recorded biomedical cause of death under primary veterinary care at UK practices participating in the VetCompass™ Programme from January 1st to December 31st, 2016. *N* = 378. *CI confidence interval **Separate categories are presented for group-level disorders with ≥ 3 affected dogs

Group-level disorder	Count	Percent (95% CI*)
Brain disorder	37	9.79 (6.79–12.78)
Kidney disorder	33	8.73 (5.88–11.58)
Appetite disorder	31	8.20 (5.44–10.97)
Heart disorder	29	7.67 (4.99–10.35)
Collapsed	25	6.61 (4.11–9.12)
Enteropathy	25	6.61 (4.11–9.12)
Neoplasia	23	6.08 (3.67–8.49)
Behavioural disorder	22	5.82 (3.46–8.18)
Lower respiratory tract disorder	20	5.29 (3.03–7.55)
Mass	18	4.76 (2.62–6.91)
Endocrine disorder	13	3.44 (1.60–5.28)
Musculoskeletal disorder	12	3.17 (1.41–4.94)
Incontinence	10	2.65 (1.03–4.26)
Spinal cord disorder	10	2.65 (1.03–4.26)
Liver disorder	9	2.38 (1.25–4.44)
Pancreatic disorder	9	2.38 (1.25–4.44)
Traumatic injury	9	2.38 (1.25–4.44)
Lethargy	8	2.12 (1.07–4.10)
Upper respiratory tract disorder	6	1.59 (0.73–3.40)
Female reproductive disorder	4	1.06 (0.41–2.67)
Ophthalmological disorder	3	0.79 (0.27–2.30)
Oral cavity disorder	3	0.79 (0.27–2.30)
Other**	19	5.03 (2.82–7.23)

## Discussion

This is the largest study to date reporting the overall disorder burden for Yorkshire Terriers based on anonymised EHR data shared from primary-care veterinary practices in the UK. The results confirm that Yorkshire Terrier remains a popular breed in the UK, although ownership levels are decreasing. Dental disease is the predominant disorder recorded, and brain disorders are the most common cause of death in Yorkshire Terriers. The longevity of Yorkshire Terriers is greater than that of dogs overall in the UK, suggesting that the breed has good overall health.

Over recent years, downstream effects related to rapidly changing consumer preferences for different dog breeds have become increasingly recognised as critical drivers of canine health and welfare issues [30]. The rapidly increasing popularity of some breeds can promote the overuse of some sires, leading to a ‘popular sire effect’ with reduced genetic diversity even within numerically large breeds [31], as well as promoting the normalisation of extreme conformations despite serious associated health and welfare problems [32, 33]. Conversely, reducing breed popularity can lead to seriously reduced genetic diversity and consequently promote new emerging genetic health issues as the available pool of breeding animals diminishes until an ultimate situation whereby justifying the continued existence of some breeds in the presence of rising disease and low ownership demand can become challenging [4, 34]. Consequently, a good understanding of overall demography is critical to protect the genetic health of dogs within each individual breed, both for the pedigree subsets registered by kennel clubs worldwide that are likely to more inbred and for the wider national population subsets that are likely to be more outbred. Although the Yorkshire Terrier was historically common among KC registrations, the pedigree dog numbers of the breed have rapidly declined in favour in recent years, decreasing by a factor of five from 0.93% of all registrations in 2013 to 0.18% of all registrations in 2022 [3]. Given this progressive decline in registrations of pedigree Yorkshire Terriers, the breed may soon join the KC list of vulnerable native breeds at risk of disappearing because they are no longer deemed desirable for ownership in the modern world or perhaps other breeds are just considered more fashionable, such as the recent surge in popularity of new designer crossbred dogs [4, 5]. However, the current study suggests an even more complex demographic pattern for the wider Yorkshire Terrier ownership in the UK. Proportional ownership of Yorkshire Terriers among the wider population of UK dogs appears to be much greater than the proportion of annual KC pedigree dogs, with the current study reporting that 3.10% of all dogs in 2016 were Yorkshire Terriers, whereas a more recent VetCompass study reported that Yorkshire Terriers represented 2.37% of all dogs in 2019 [5]. The current study also revealed a much shallower proportional decline in new Yorkshire Terrier puppies entering the wider UK dog population, decreasing from 3.54% in 2005 to 2.15% in 2016. This suggests that the move away from Yorkshire Terriers by UK owners overall is not as dramatic as the KC registrations suggest and that it is specifically the pedigree subset of Yorkshire Terriers bred to meet the KC breed standards that truly is suffering the greatest decline. The KC breed standard places a maximum bodyweight of 3.2 kg for the Yorkshire Terrier and now allocates the breed to the Toy group of KC breeds rather than the Terrier group despite the breed’s name [2]. However, analysis of the bodyweight data from the current study revealed a substantially greater median adult bodyweight of 5.06 kg for the wider UK population of Yorkshire Terriers, with 75% of these dogs weighing more than 3.81 kg. There is increasing debate over health and welfare impacts and opportunities from the growing genetic and phenotypic divergence between the registered pedigree subsets of dogs required to meet strict KC parentage rules and breed standards that change only slowly over time, compared with the wider population of unregistered dogs unencumbered by such rules and where change can be much faster [35]. In the case of the Yorkshire Terrier, it may be that relaxing the KC body size limitation and promoting outcrossing to generate a new, larger and perhaps physically more robust type of Yorkshire Terrier dog that the public prefers could rescue the pedigree subset of this breed from reaching the end of its current apparent terminal decline in ownership.

Periodontal disease is recognised as one of the most common disorders in dogs overall. A study of 22,333 dogs under primary veterinary care in the UK reported periodontal disease, with a prevalence of 12.52%, as the most commonly diagnosed disorder in UK dogs [22]. In the present study, periodontal disease was similarly the disease most commonly diagnosed in Yorkshire Terriers, with a much higher prevalence of 21.10%, that is almost twice as high as the overall prevalence in dogs. These results suggest that periodontal disease should be considered an important disorder predisposition in Yorkshire Terriers. This conclusion is supported by previous work on dogs under primary veterinary care in the UK, which reported a similarly high prevalence of 22.22% for periodontal disease in the Yorkshire Terrier [36]. However, that previous study also revealed aging as a strong risk factor for periodontal disease in dogs, which was supported by the increasing prevalence of dental disease with age identified in Fig. 3 of the current study, and which could partially explain the high prevalence in Yorkshire Terriers as a long-lived breed overall. However, even after accounting for age in multivariable analysis, the Yorkshire Terrier in that previous study still had 2.16 times greater odds of periodontal disease than crossbred dogs did [36]. That study also identified small body size as a strong risk factor for periodontal disease, with dogs weighing less than 10 kg having 3.07 times greater odds than dogs weighing 30–40 kg. The typically small body size of the Yorkshire Terrier breed could also lead to challenges for owners to brush their teeth, along with greater reluctance in small dogs to gnaw on dental chews and a reputation for fussy eating habits [37]. Awareness of both a high prevalence and a predisposition to periodontal disease in Yorkshire Terriers could be used to encourage owners to undertake greater daily preventative home care [38]. These opportunities for improved dental health could include tooth brushing [39], dental solutions [40], bones and chews [41], active ingredients (e.g., chlorhexidine [42]) or polyphosphates [43]), and dental diets [44].

Persistent deciduous teeth are defined as deciduous teeth that remain in the mouth at the same time as their permanent counterparts have erupted [45]. The present study identified persistent deciduous teeth as a commonly diagnosed specific dental issue in Yorkshire Terriers, with a 3.57% prevalence, making this the fifth most commonly diagnosed disorder. In contrast, the overall prevalence of persistent deciduous teeth in dogs has previously been reported to be less than one third of this value, with a prevalence of 1.01%, which makes it the 29th most commonly diagnosed disorder in dogs overall [22]. Correct dentition in dogs is critical for overall health by assisting with eating, carrying and holding objects, playing and grooming [46]. As diphyodonts with two sets of teeth erupting over time, the full complement of 28 deciduous teeth in dogs starts to be replaced at approximately three months of age to achieve a full complement of 42 permanent teeth by seven months [46, 47]. However, deciduous teeth can persist when an abnormal eruption path or complete absence of a permanent tooth fails to pressure the apex of the deciduous tooth to trigger natural root-end resorption of that tooth [48]. Orthodontic issues are reported to begin as early as two weeks after the permanent canines start to erupt, whereby the persistent deciduous tooth occupies and blocks the correct section of the periodontium from attaching to the permanent tooth. Therefore, persistent deciduous teeth are recommended to be extracted as early as possible [49]. Awareness of the heightened risk of persistent deciduous teeth in Yorkshire Terriers can be used to encourage owners to monitor their dogs carefully for correct dental eruption and to pursue remedial treatment early in the case of problems being identified [45].

Patellar luxation describes a medial or lateral dislocation of the patella from the trochlear groove of the femur and can be either developmental or traumatic, although most cases are considered developmental [50]. Patellar luxation predisposes individuals to later stifle joint osteoarthritis, with welfare impacts linked to chronic pain and reduced freedom to express the natural canine need for exercise [51]. In the present study, Yorkshire Terriers had a 3.42% prevalence of patellar luxation, which is substantially higher than the 1.04% prevalence reported previously in dogs overall in the UK [22]. Predisposition to patellar luxation is supported by an earlier study of dogs under primary veterinary case in England that reported that the Yorkshire Terrier had 5.5 times greater odds of patellar luxation than crossbred dogs, with this predisposition being exceeded only by the Pomeranian (x 6.5 times greater odds) and the Chihuahua (x 5.9 times greater odds) [52]. An international analysis of pet insurance records on 600,000 dogs in Sweden also revealed a strong predisposition, with Yorkshire Terriers showing a 7.3-fold greater relative risk for patellar luxation than all other insured dogs [53]. The aetiopathogenesis of patellar luxation is still poorly understood, with some genetic factors implicated, but a major contribution seems to come from multiple skeletal abnormalities that affect overall hindlimb alignment [50, 54]. Very small types of dogs appear heavily predisposed, so there may be value in breeding away from extremes of miniaturisation in typically small body size breeds [53, 55–57]. Several different surgical approaches to mitigate some of the longer term sequelae have been described, so raising awareness early for owners of Yorkshire Terriers about heightened risk of patellar luxation can support later discussions on the most appropriate forms of veterinary care [50].

Across humans and those populations of animals where life is aimed to extend to either an unassisted death or to euthanasia on welfare grounds, low longevity is taken to reflect a high cumulative burden of events leading to mortality and therefore to indicate a generally less healthy population, with high longevity conversely indicating good overall health [11]. In the present study, the median age at death for Yorkshire Terriers was 13.56 years. This longevity is over 18 months longer than the 12.0-year median longevity previously reported for dogs overall in the UK via a methodology similar to that used in the current study [12]. Similarly, an analysis of mortality data from over 30,000 UK dogs that created life tables for 18 common dog breeds reported the Yorkshire Terrier with a 12.54-year life expectancy that was the second highest breed life expectancy from the first year of life, surpassed only by the Jack Russell Terrier at 12.72 years [6]. International data also suggest high longevity in Yorkshire Terriers in other countries. An analysis of mortality data on 4,957 deaths in dogs in Italy revealed that Yorkshire Terrier, with a median longevity of 10.0 years, was the highest among 38 common breeds [58]. With respect to the pedigree subset of dogs, an owner survey that included mortality data on 15,881 deaths in UK dogs registered with the KC reported a median longevity of 12.67 years for the Yorkshire Terrier. This longevity was greater than the 11.25 years reported for the pedigree dogs overall, suggesting that comparatively high longevity also exists for the physically smaller and more inbred pedigree subset of the breed [13]. In all of these longevity studies that covered multiple breeds, the lifespan of the Yorkshire Terrier compared very favourably against the lifespan of other breeds, even those with similarly small body size. This overall body of evidence on high longevity supports a view of the Yorkshire Terrier breed overall as a largely healthy and robust type of dog, although these conclusions need to be tempered with the caveat that small dogs, such as Yorkshire Terrier, generally have an overall longevity advantage over large dogs [6, 12, 59]. In addition, it should also be noted that there remains discussions about whether extended lifespan can always be safely interpreted as meaning extended healthspan (i.e. that proportion of overall lifespan that is deemed to be lived in good health) equally across all dog breeds [60, 61]. Aging itself is a very strong risk factor for a wide range of degenerative, inflammatory and neoplastic disorders that can dramatically reduce quality of life [62].

The current study had several limitations. This study reports on disorder prevalence to provide results on absolute disorder risk but does not analytically compare risk between Yorkshire Terriers and all remaining dogs under veterinary care from the same underlying denominator dog population [63]. While descriptive prevalence studies such as the current work are useful, uncertainty and the risk of bias are introduced when inferring predisposition by comparing prevalence results between studies carried out using different methods and sources of denominator populations. Although the current study reports on disorder prevalence and uses this information to consider the wider welfare impact, a fuller welfare impact assessment would require additional data on disorder duration and severity that were not extracted within the current study [64]. The current study reports apparent disorder prevalence in terms of the frequency of annual diagnosis under primary veterinary care, but the proportion of all true cases that receive a formal diagnosis may often be substantially less than 100%, especially for disorders that are complex or expensive to diagnose; therefore, the current ‘apparent prevalence’ results may underestimate ‘true disorder prevalence’ [65]. The current results reflect the factual proportions of Yorkshire Terriers formally diagnosed with each of the disorders included but the relative proportion of the propensity to each of these disorders that can be ascribed to intrinsic risk related to the individual dogs compared to the extrinsic risk related to the dog’s environment or owner remains unknown [66].

## Conclusions

The current study revealed reducing ownership levels for Yorkshire Terriers in the UK, although this decline appears to be steeper in the pedigree subset of dogs that are physically smaller and likely more inbred than in the wider Yorkshire Terrier population. Disorders that are common and appear predisposed in Yorkshire Terriers include periodontal disease, persistent deciduous teeth and patellar luxation. Overall, Yorkshire Terrier has high longevity, which is suggestive of robust overall health.

## Data Availability

The dataset supporting the conclusions of this article is available at Figshare 10.6084/m9.figshare.25573914.

## Abbreviations

BCS: Body condition score
CI: Confidence interval
EHR: Electronic health record
IQR: Interquartile range
KC: Kennel Club
